# Widespread Parenchymal Abnormalities and Pulmonary Embolism on Contrast-Enhanced CT Predict Disease Severity and Mortality in Hospitalized COVID-19 Patients

**DOI:** 10.3389/fmed.2021.666723

**Published:** 2021-06-29

**Authors:** Francesca Campoccia Jalde, Mats O. Beckman, Ann Mari Svensson, Max Bell, Magnus Sköld, Fredrik Strand, Sven Nyren, Anna Kistner

**Affiliations:** ^1^Department of Anesthesiology, Surgical Services and Intensive Care, Karolinska University Hospital, Stockholm, Sweden; ^2^Department of Molecular Medicine and Surgery, Karolinska Institutet, Stockholm, Sweden; ^3^Department of Radiology, Solna, Karolinska University Hospital, Stockholm, Sweden; ^4^Department of Physiology and Pharmacology, Karolinska Institutet, Stockholm, Sweden; ^5^Respiratory Medicine Unit, Department of Medicine Solna and Center for Molecular Medicine, Karolinska Institutet, Stockholm, Sweden; ^6^Department of Respiratory Medicine and Allergy, Karolinska University Hospital Solna, Stockholm, Sweden; ^7^Department of Oncology-Pathology, Karolinska Institutet, Stockholm, Sweden; ^8^Medical Radiation Physics and Nuclear Medicine, Karolinska University Hospital, Stockholm, Sweden

**Keywords:** C-reactive protein, pulmonary thromboembolism, computed tomography, pulmonary trunk, critical care, mortality

## Abstract

**Purpose:** Severe COVID-19 is associated with inflammation, thromboembolic disease, and high mortality. We studied factors associated with fatal outcomes in consecutive COVID-19 patients examined by computed tomography pulmonary angiogram (CTPA).

**Methods:** This retrospective, single-center cohort analysis included 130 PCR-positive patients hospitalized for COVID-19 [35 women and 95 men, median age 57 years (interquartile range 51–64)] with suspected pulmonary embolism based on clinical suspicion. The presence and extent of embolism and parenchymal abnormalities on CTPA were recorded. The severity of pulmonary parenchymal involvement was stratified by two experienced radiologists into two groups: lesions affecting ≤50% or >50% of the parenchyma. Patient characteristics, radiological aspects, laboratory parameters, and 60-day mortality data were collected.

**Results:** Pulmonary embolism was present in 26% of the patients. Most emboli were small and peripheral. Patients with widespread parenchymal abnormalities, with or without pulmonary embolism, had increased main pulmonary artery diameter (*p* < 0.05) and higher C-reactive protein (*p* < 0.01), D-dimer (*p* < 0.01), and troponin T (*p* < 0.001) and lower hemoglobin (*p* < 0.001). A wider main pulmonary artery diameter correlated positively with C-reactive protein (*r* = 0.28, *p* = 0.001, and *n* = 130) and procalcitonin. In a multivariant analysis, D-dimer >7.2 mg/L [odds ratio (±95% confidence interval) 4.1 (1.4–12.0)] and ICU stay were significantly associated with embolism (*p* < 0.001). The highest 60-day mortality was found in patients with widespread parenchymal abnormalities combined with pulmonary embolism (36%), followed by patients with widespread parenchymal abnormalities without pulmonary embolism (26%). In multivariate analysis, high troponin T, D-dimer, and plasma creatinine and widespread parenchymal abnormalities on CT were associated with 60-day mortality.

**Conclusions:** Pulmonary embolism combined with widespread parenchymal abnormalities contributed to mortality risk in COVID-19. Elevated C-reactive protein, D-dimer, troponin-T, P-creatinine, and enlarged pulmonary artery were associated with a worse outcome and may mirror a more severe systemic disease. A liberal approach to radiological investigation should be recommended at clinical deterioration, when the situation allows it. Computed tomography imaging, even without intravenous contrast to assess the severity of pulmonary infiltrates, are of value to predict outcome in COVID-19. Better radiological techniques with higher resolution could potentially improve the detection of microthromboses. This could influence anticoagulant treatment strategies, preventing clinical detoriation.

## Introduction

Severe acute respiratory syndrome coronavirus-2 (SARS-CoV-2) spread in Europe in March 2020, peaking in Sweden in the middle of April 2020. A new wave of infection then struck the country in the late fall of 2020 and in the spring of 2021.The infection syndrome has been named COVID-19.

SARS-CoV-2 virus infects epithelial cells in human airways through the interaction between the viral S protein and the angiotensin converting enzyme 2 (ACE2) receptor, causing severe pneumonia in some patients (1). COVID-19 and the associated pneumonia leads to typical findings on computed tomography (CT) (2). In COVID-19, elevated D-dimer, which may indicate thrombus formation and degradation, is associated with higher mortality (3). A high frequency of thrombosis and pulmonary embolism (PE) has been reported in hospitalized COVID-19 patients (4–6). It has been suggested that in COVID-19 acute respiratory distress syndrome (ARDS), the virus may induce pulmonary endothelial microvascular damage, which may also lead to microthrombosis (7).

We hypothesized that PE, systemic inflammation, and widespread parenchymal abnormalities (WPA) would affect survival in COVID-19. To test this hypothesis, we studied hospitalized COVID-19 patients with suspected PE and the relationship between abnormal radiological and laboratory findings and the risk of fatal outcome at pandemic inception. Such information could be useful for early identification of patients at risk of thromboembolic events who require further radiological investigation.

## Patients and Methods

### Study Design

The study was approved by the Swedish Ethical Review Authority (application number 2020-01882), and waived informed consent was obtained. From March 19 to May 31, 2020, we included all patients with a positive reverse transcription polymerase chain reaction (RT-PCR) SARS-CoV-2 test who were referred for computed tomography pulmonary angiogram (CTPA) due to clinically suspected PE (sudden clinical deterioration, declining PaO_2_/FiO_2_, echocardiographic indirect signs of pulmonary hypertension, and dilated right ventricle). Patients were enrolled prospectively, by identifying them in the hospital radiological database system [hospital information system (HIS) and radiological information system (RIS)/picture archiving and communication system (PACS)].

Patients with PE on CTPA were stratified according to the radiological picture of embolization. Information on the duration of symptoms, hospital and intensive care unit (ICU) stay, mechanical ventilation, and comorbidities were collected.

Laboratory results for blood parameters were collected on the day of CTPA. The highest concentration of C-reactive protein (CRP) during the hospital stay was also recorded. We also collected results from the following routine analyses when available: NT-proBNP (*n* = 66) and PaO_2_/FiO_2_ (designated PFI) on the day of the CTPA and the lowest value during hospitalization (*n* = 66). Demographics, including age, sex, weight, length, body mass index, comorbidities, and 60-day mortality after first symptoms, were recorded, as well as anti-thrombotic and steroid treatment. Mortality incidence was retrieved from journal records, which is connected to the population registration authority in Sweden.

### Patients

During the study period, 640 patients were hospitalized at Karolinska University Hospital, Solna, with a documented SARS-CoV-2 infection. Starting from April 3, COVID-19 patients hospitalized in the wards received thrombosis prophylaxis with low-molecular-weight heparin (LMWH) [Tinzaparin 3500–4500 U, subcutaneous (SC) once daily (OD) or Dalteparin 5000 U SC OD, adjusted for weight and kidney function], according to general advice from Stockholm Health Care Region. All ICU patients received standard thrombosis prophylaxis from the start of the study, but from April 14, the LMWH dose was intensified and patients were given an intermediate dose (in normal weight patients, 5000 U SC twice daily) (8). A few patients had therapeutic LMWH at the time of CTPA. In addition, five patients received oral apixaban (5 mg bid) and two patients received continuous unfractionated heparin. Selected patients were provided prednisone (2.5–40 mg/day) or betamethasone (2–24 mg/day).

**Figure 1:**
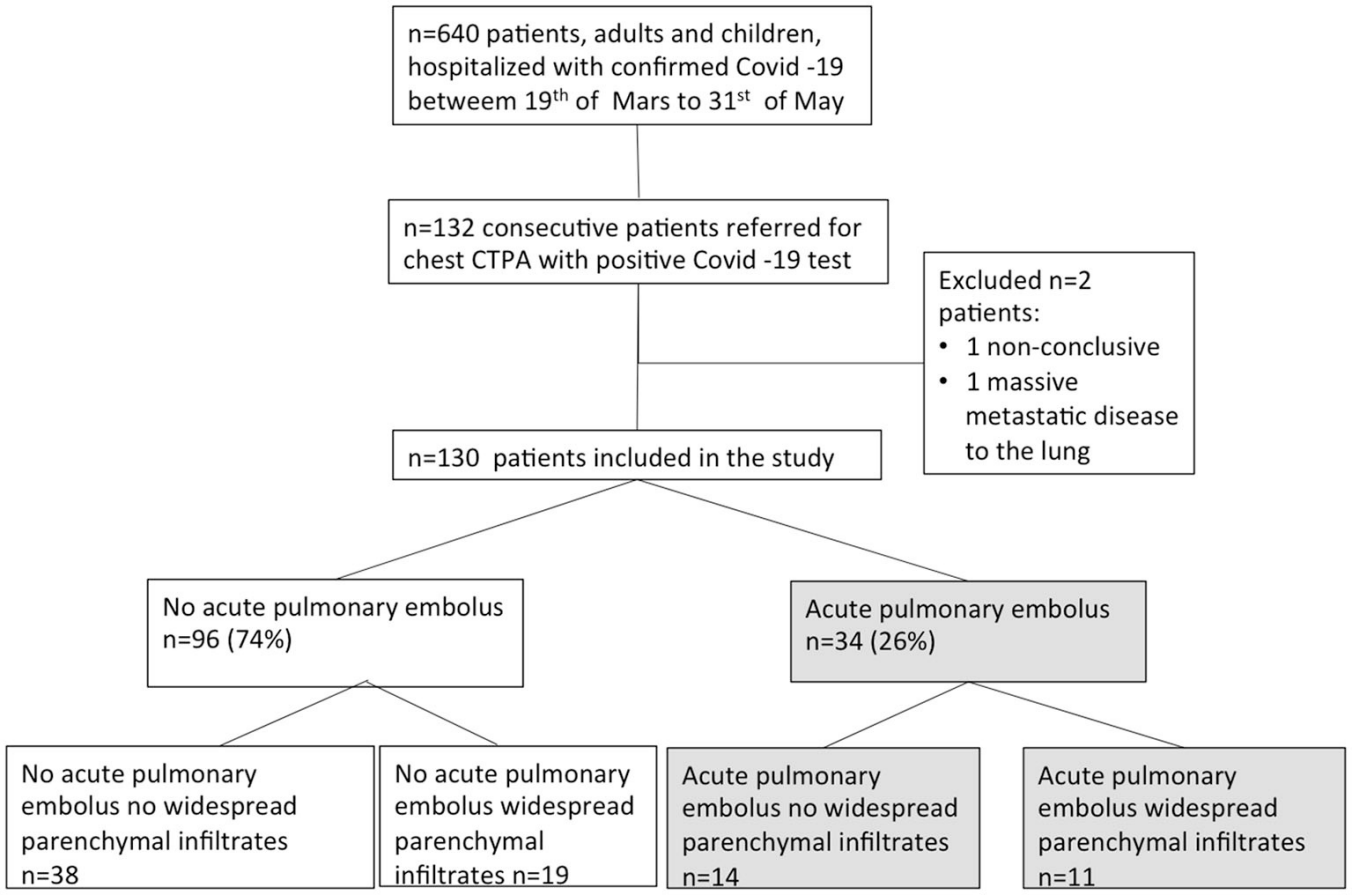
Flowchart of patient inclusion.

Patients who underwent chest CT without intravenous contrast or with contrast only in venous phase were excluded, as well as a few subjects with CTPA and suspected COVID-19, but with a negative SARS-CoV-2 test. Two SARS-CoV-2-positive patients with CTPA were excluded because of impossible interpretation of the CT and massive metastatic lung disease (Figure 1).

### CT Protocol

A 256-slice multi-detector CT (Revolution CT, GE Healthcare, Milwaukee, Wisconsin, USA) was used for CTPA after intravenous injection of 60 ml of iodinated contrast agent (Johexol 350 mg/ml) with a flow rate of 5 ml/s. The contrast agent dose was slightly reduced in renal insufficiency. For older patients, scanning was triggered on the main pulmonary artery. The dose-length product ranged 90–300 milligray^*^cm and the CT dose-index volume was typically 4–10 milligray^*^cm, peaking at 15 milligray^*^cm in some cases with high BMI.

**Figure 2:**
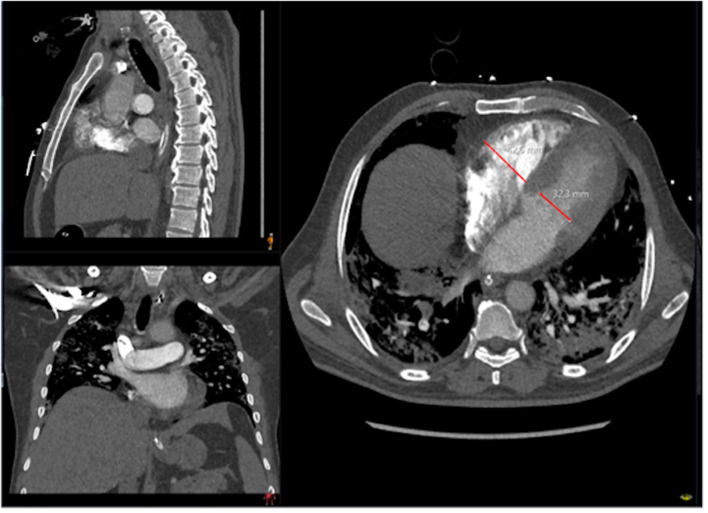
Assessment of the right and left ventricular diameter in axial four-chamber view. The red lines show where the measurements were performed.

**Figure 3:**
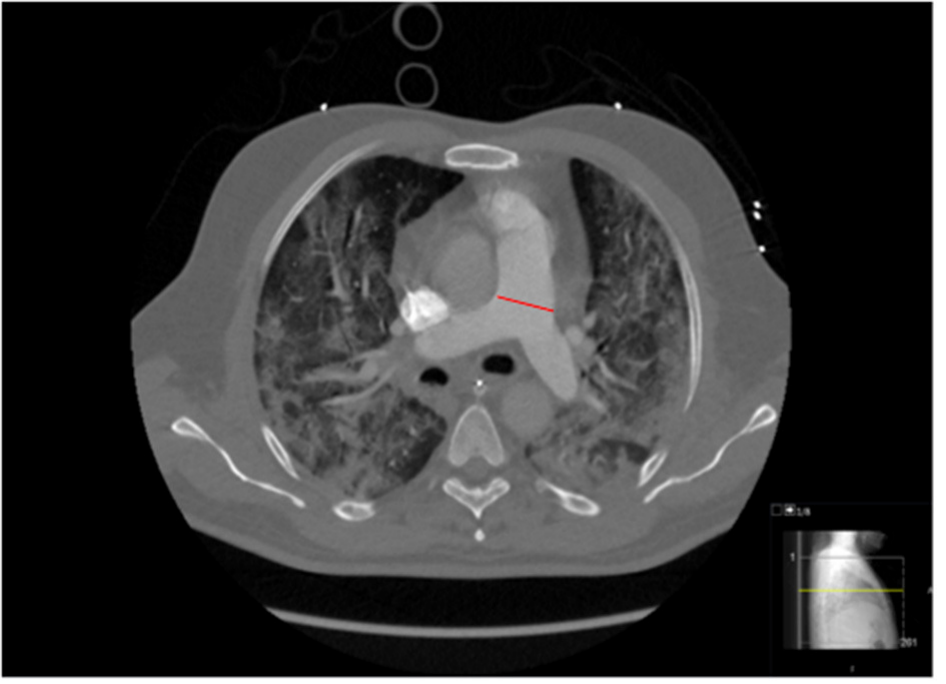
Assessment of main artery pulmonary diameter on axial view. The red line indicates where the diameter was measured.

The presence of pulmonary emboli was analyzed independently by two experienced emergency radiologists on a PACS workstation (SECTRA AB), using 0.63-mm slice reformats in orthogonal planes. Slices classified as negative for PE were analyzed again by a specialist in thoracic radiology. CT scans positive for PE were categorized according to the Qanadli score. This index quantifies the extension of the arterial obstruction on CTPA imaging (9). Right ventricular diameter was estimated using 0.63-mm slice reformats in the axial plane in four-chamber view (Figure 2), and severe right ventricular dysfunction (right/left ventricular quotient >1.3) was estimated. The main pulmonary artery diameter was measured just proximal to the pulmonary artery bifurcation, on an axial slice perpendicular to its long axis, using 2-mm slice formats (Figure 3). Data on patients with and without PE are presented in Table 1.

**Table 1:** Demographics, comorbidities, radiology, laboratory, and outcome data for hospitalized COVID-19 individuals with or without PE undergoing CTPA (*n* = 130).

**Demographics**	**Pulmonary embolism**	**No pulmonary embolism**	***p*-value**
	**(*n* = 34)**	**(*n* = 96)**	
Gender (male/female)	27/7	68/28	0.33^†^
Age (years), *n* = 130	58 (51–64)	58 (50–65)	0.85
Weight (kg), *n* = 121	82 (71–95)	81 (72–94)	0.76
Length (cm), *n* = 114	174 (171–180)	173 (165–181)	0.54
BMI (kg/m^2^), *n* = 113	27 (23–29)	28 (24–31)	0.80
**Anticoagulant therapy**			
24 h before CT scan	76 (*n* = 26/34)	74 (*n* = 71/96)	0.86^†^
Prophylactic (Standard)	9 (*n* = 3/34)	33 (*n* = 32/95)	
Intermediate	53 (*n* = 18/34)	28 (*n* = 27/95)	0.016^†^
Therapeutic	15 (*n* = 5/34/	13 (*n* = 12/95)	
**Corticosteroid treatment**			
Corticosteroid at CT scan	6 (*n* = 2/34)	18 (*n* = 17/79)	0.09^†^
**Comorbidities**			
Time from symptoms to CT (days)	23 (15–33)	17 (11–26)	0.014
Time from hospital admission to CT (days)	14 (4–20)	6 (1–15)	0.07
Hypertension	44 (*n* = 14/32)	36 (*n* = 34/94)	0.45^†^
Hyperlipidemia	18 (*n* = 6/33)	11 (*n* = 10/95)	0.25^†^
Previous AMI	6 (*n* = 2/34)	8 (*n* = 7/96)	0.64^†^
Heart failure	0 (*n* = 0/34)	7 (*n* = 7/96)	0.11^†^
Diabetes	12 (*n* = 4/34)	19 (*n* = 18/95)	0.34^†^
COPD	3 (*n* = 1/32)	5 (*n* = 5/96)	0.63^†^
OSAS	9 (*n* = 3/34)	3 (*n* = 3/95)	0.18^†^
Asthma	22 (*n* = 7/32)	16 (*n* = 15/96)	0.42^†^
Kidney disease	3 (*n* = 1/34)	4 (*n* = 4/96)	0.75^†^
Thromboembolic disease	0 (*n* = 0/34)	1 (*n* = 1/96)	0.55^†^
Cancer (active and/or cured)	12 (*n* = 4/33)	15 (*n* = 14/95)	0.71^†^
**Radiologic morphology**			
Widespread parenchymal abnormalities	65 (*n* = 22/34)	56 (*n* = 54/96)	0.39^†^
Main pulmonary artery diameter (mm), *n* = 130	29.3 (27.5–31.1)	28.5 (27.6–29.4)	0.43
Right ventricular diameter (mm), *n* = 130	41.1 (38.3–43.9)	38.5 (37.2–39.9)	0.06
Severe RV dysfunction on CT scan (RV/LV > 1.3)	18 (*n* = 13/34)	14 (*n* = 13/96)	0.56^†^
**Laboratory data**			
CRP maximum during hospitalization (mg/L), *n* = 130	295 (250–341)	214 (187–241)	0.003
CRP at CT scan (mg/L), *n* = 130	149 (104–194)	102 (83–121)	0.026
White blood cell count at CT scan (10^9^/L), *n* = 130	12.1 (10.1–14.2)	9.4 (8.3–10.4)	<0.01
Hemoglobin at CT scan (g/L), *n* = 130	105 (98–112)	117 (113–121)	0.004
Platelet count (10^9^/L)	334 (268–399)	340 (307–373)	0.85
P-creatinine at CT scan (μmol/L), *n* = 130	107 (82–131)	78 (69–87)	0.007
P-procalcitonin (μg/L), *n* = 128	0.59 (0.30–1.17)	0.33 (0.24–0.47)	0.10
D-dimer at CT scan (mg/L), *n* = 122	8.2 (5.8–11.8)	2.1 (1.6–2.7)	<0.0001
Troponin T at CT scan (ng/L), *n* = 110	41 (28–61)	18 (14–23)	0.0004
NT-proBNP at CT scan (ng/L), *n* = 66	1,410 (620–3200)	750 (410–1360)	0.20
Lowest PFI (PaO_2_/FiO_2_), *n* = 62	12.1 (9.7–14.6)	11.8 (9.8–13.8)	0.70^††^
PFI at CT scan (PaO_2_/FiO_2_), *n* = 56	20.8 (14.9–26.6)	15.7 (12.8–18.6)	0.25^††^
**Outcome data**			
Hospitalized at the end of study	21 (*n* = 7/34)	16 (*n* = 15/96)	0.51^†^
Intubated at CT scan	56 (*n* = 19/34)	25 (*n* = 24/96)	0.001^†^
ICU at CT scan	56 (*n* = 19/34)	24 (*n* = 23/96)	<0.001^†^
Vasopressor treatment at CT scan	64 (*n* = 21/33)	32 (*n* = 29/92)	0.001^†^
Renal replacement therapy at CT scan	38 (*n* = 13/34)	13 (*n* = 12/96)	0.001^†^
ICU during stay	85 (*n* = 29/34)	45 (*n* = 43/96)	<0.0001^†^
30-day mortality	24 (*n* = 8/34)	15 (*n* = 14/96)	0.23^†^
60-day mortality	29 (*n* = 10/34)	15 (*n* = 14/96)	0.06^†^
Anticoagulant therapy at CT in deceased patients	70 (*n* = 7/10)	86 (*n* = 12/14)	0.35^†^

*Demographics are expressed as median (interquartile range). Radiological and laboratory data are expressed as mean (± CI). Procalcitonin, D-dimer, troponin T, and NT-ProBNP were not normally distributed and log-transformed and are expressed as geometric mean (± CI). Comorbidities, radiological, morphology, and outcome data are expressed as %, n, affected subjects/total subjects in each group*.

*Statistical analyses are based on analysis of variance (t-test) between groups. p <0.05 was considered significant*.

† *Pearson chi-squared*.

†† *Values were log-transformed before calculating. AMI, acute myocardial infarction; BMI, body mass index; CI, confidence interval; COPD, chronic obstructive pulmonary disease; CRP, C-reactive protein; CT, computed tomography; CTPA, computed tomography pulmonary artery; ICU, intensive care unit; N.A., not applicable; PE, pulmonary embolism; OSAS, obstructive sleep apnea syndrome; PFI, quotient between arterial partial oxygen pressure and the fraction of inspired oxygen; NT-proBNP, N-terminal pro brain natriuretic peptide; RV, right ventricular*.

**Figure 4:**
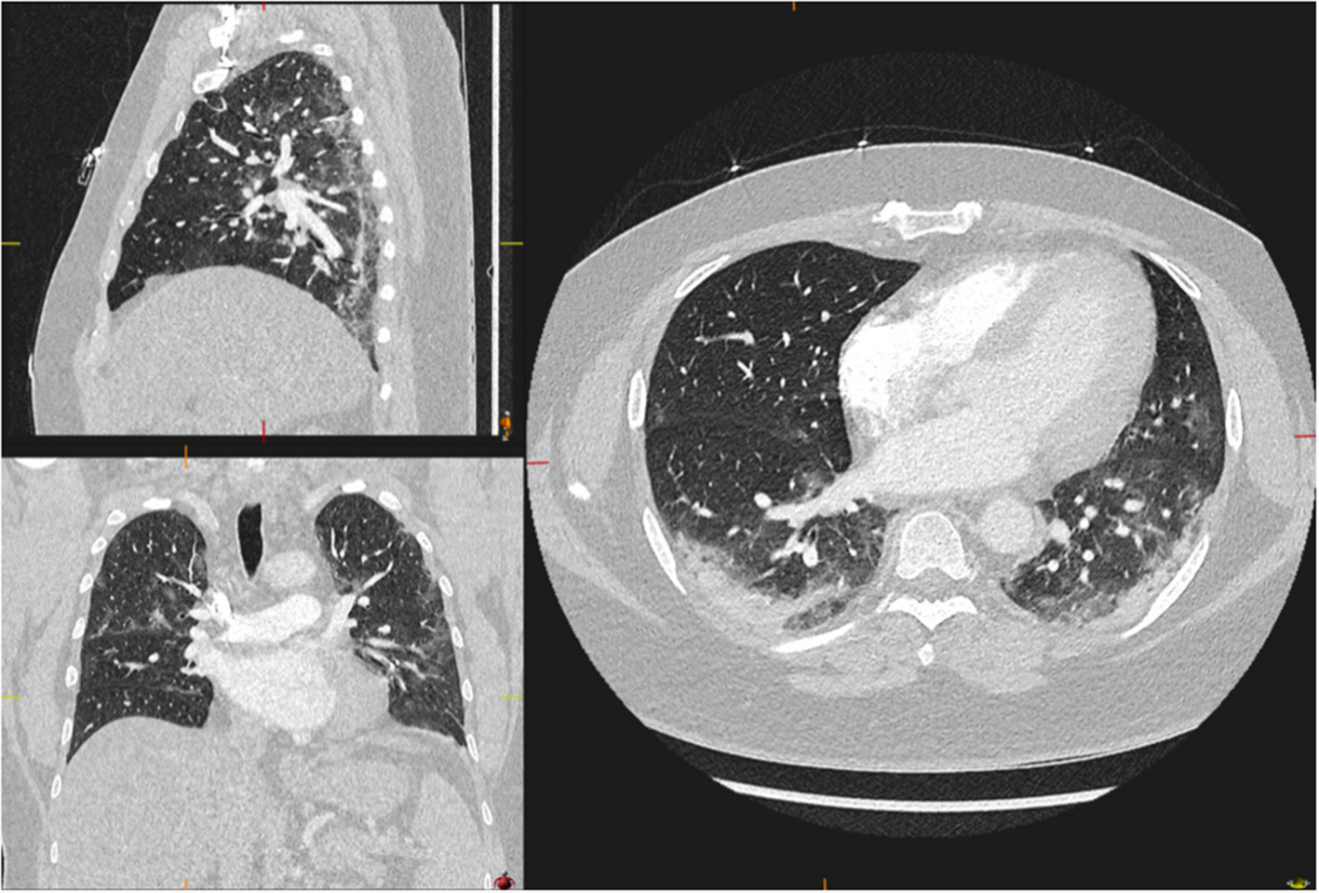
Example of lung parenchyma involvement. A 46-year-old male with no widespread parenchymal abnormalities (infiltrates affecting <50% of the lungs). Forty-two percent of the whole cohort was classified as having <50% involvement of the parenchyma.

**Figure 5:**
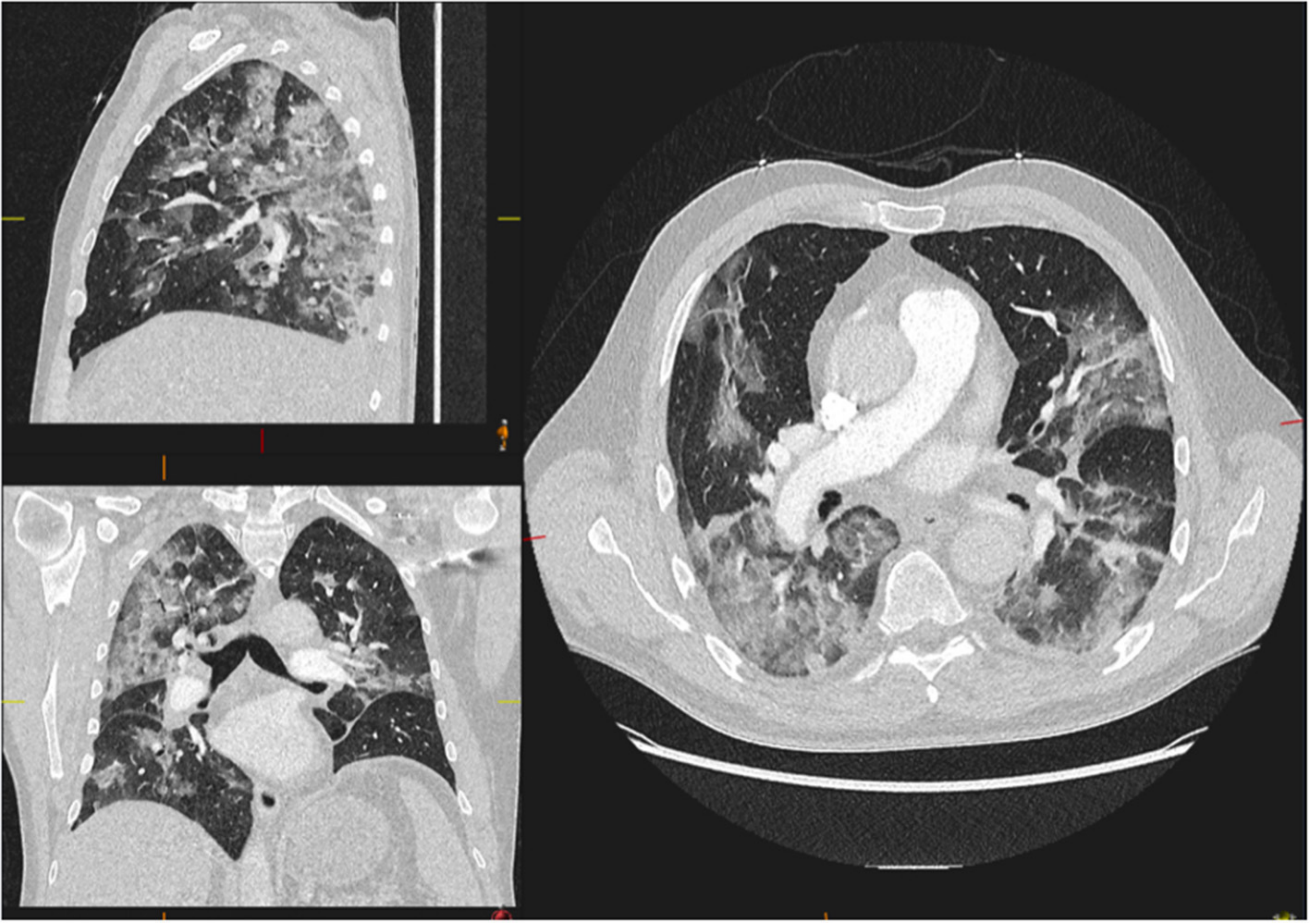
Examples of lung parenchyma involvement. A 62-year-old male with widespread parenchymal abnormalities (infiltrates affecting >50% of the lungs). Fifty-eight percent of the whole cohort was classified as having >50% involvement of the parenchyma.

The severity of pulmonary parenchymal involvement was stratified according to the extension of parenchymal abnormalities (e.g., ground-glass opacities, crazy-paving appearance, airspace consolidations, broncho-vascular thickening, and traction bronchiectasis) (10). WPA was defined as lesions that were spread over more than 50% of the parenchyma affecting all lung lobes. All other patterns were classified as not WPA, modified from Revel et al. (11) (Figures 4, 5). Two specialists in thoracic radiology performed the classification in double-blind. In patients where the radiologists' opinions differed (13 out of 130 subjects, 10%), an additional classification was performed together to reach consensus. The WPA and no-WPA angiograms were then further subgrouped according to the presence or absence of PE (Table 2).

**Table 2:** Demographics, comorbidities, radiology, laboratory, and outcome data of COVID-19 individuals with or without PE divided into presence or absence of widespread parenchymal abnormalities.

**Demographics**	**1.Not widespread parenchymal abnormalities, no PE**	**2. Widespread parenchymal abnormalities, no PE**	**3. Not widespread parenchymal abnormalities + PE**	**4. Widespread parenchymal abnormalities + PE**	***p*-value**
	**(*n* = 43)**	**(*n* = 53)**	**(*n* = 12)**	**(*n* = 22)**	
Gender (male/female)	28/15	40/13	10/2	17/5	0.50^†^
Age (years)	58 (49–65)	57 (51–64)	58 (48–63)	58 (51–68)	0.92
Weight (kg)	80 (72–94)	82 (72–93)	82 (69–93)	83 (72–97)	0.92
Length (cm)	177 (164–183)	172 (165–179)	172 (170–183)	175 (172–180)	0.49
BMI (kg/m^2^)	27 (24–31)	28 (24–31)	29 (23–30)	27 (23–29)	0.99
**Anticoagulant therapy**					
24 h before CT scan	60 (*n* = 26/43)^*^ vs. 3	85 (*n* = 45/53)^*^ vs. 3	58 (*n* = 7/12)	86 (*n* = 19/22)^*^ vs. 3	0.007^†^
Prophylactic (Standard)	37 (*n* = 16/43)	30 (*n* = 16/52)	17 (*n* = 2/12)	5 (*n* = 1/22)	
Intermediate	19 (*n* = 8/43	36 (*n* = 19/52)	17 (*n* = 2/12)	73 (*n* = 16/22)	<0.001^†^
Therapeutic	5 (*n* = 2/43)	19 (*n* = 10/52)	25 (*n* = 3/12)	9 (*n* = 2/22)	
**Corticosteroid treatment**					
Corticosteroid at CT scan	16 (*n* = 7/43)	20 (*n* = 10/49)	8 (*n* = 1/12)	5 (*n* = 1/22)	0.32^†^
**Comorbidities**					
Time from symptoms to CT (days)	14 (12–20)	23 (11–31)^**^ vs. 1	19 (14–30)	25 (16–36)^***^ vs. 1	<0.001
Time from hospital admission to CT (days)	3 (1–6)	13 (3–22)^***^ vs. 1	12 (2–19)* vs. 1	16 (4–22)^***^ vs. 1	<0.0001
Hypertension	33 (*n* = 14/42)	38 (*n* = 20/52)	42 (*n* = 5/12)	45 (*n* = 9/20)	0.83^†^
Hyperlipidemia	12 (*n* = 5/43)	10 (*n* = 5/52)	18 (*n* = 2/11)	18 (*n* = 4/22)	0.71^†^
Previous AMI	2 (*n* = 1/43)	13 (*n* = 7/53)	0 (*n* = 0/12)	9 (*n* = 2/22)	0.17^†^
Heart failure	7 (*n* = 3/43)	8 (*n* = 4/53)	0 (*n* = 0/12)	0 (*n* = 0/22)	0.45^†^
Diabetes	14 (*n* = 6/42)	23 (*n* = 12/53)	8 (*n* = 1/12)	14 (*n* = 3/22)	0.53^†^
COPD	5 (*n* = 2/43)	6 (*n* = 3/53)	8 (*n* = 0/12)	5 (*n* = 1/22)	0.87^†^
OSAS	5 (*n* = 2/42)	2 (*n* = 1/53)	8 (*n* = 1/12)	9 (*n* = 2/22)	0.52^†^
Asthma	14 (*n* = 6/43)	17 (*n* = 9/53)	27 (*n* = 3/11)	19 (*n* = 4/21)	0.76^†^
Kidney disease	0 (*n* = 0/43)	8 (*n* = 4/53)	8 (*n* = 1/12)	0 (*n* = 0/22)	0.16^†^
Thromboembolic disease	2 (*n* = 1/43)	0 (*n* = 0/53)	0 (*n* = 0/12)	0 (*n* = 0/22)	0.56^†^
Cancer (active and/or cured)	21 (*n* = 9/42)	9 (*n* = 5/53)	17 (*n* = 2/12)	10 (*n* = 2/21)	0.35^†^
**Radiology**					
Main pulmonary artery diameter (mm), *n* = 130	27.4 (26.0–28.8)	29.4 (28.1–30.8) ^*^ vs. 1	27.4 (24.3–30.4)	30.4 (28.1–32.6)^*^ vs. 1	0.048
Right ventricular diameter (mm), *n* = 130	38.0 (35.8–40.1)	39.0 (37.3–40.7)	39.8 (33.4–46.1)	41.9 (38.9–44.9)	0.20
Severe RV dysfunction on CT scan (RV/LV > 1.3)	12 (5/43)	15 (8/53)	8 (1/12)	23 (5/22)	0.60
**Laboratory data**					
CRP maximum during hospitalization (mg/L), *n* = 130	139 (103–174)	275 (243–307)^***^ vs. 1	241 (160–323)	325 (270–380)^***^ vs. 1/^*^ vs. 3	<0.0001
CRP at CT scan (mg/L), *n* = 130	76 (55–98)	123 (94–152)^*^ vs. 1	116 (31–201)	167 (111–222)^***^ vs. 1	0.009
White blood cell count at CT scan (10^9^/L), *n* = 130	7.1 (6.0–8.3)	11.2 (9.7–12.6)^***^ vs. 1	13.7 (9.9–17.5)^***^ vs. 1	11.3 (8.7–13.9) ^**^ vs. 1	<0.0001
Hemoglobin at CT scan (g/L), *n* = 130	125 (120–131)	109 (104–114)^***^ vs. 1	119 (105–133)	97 (90–104)^***^ vs. 1/^**^ vs. 2/^**^ vs. 3	<0.0001
P-creatinine at CT scan (μmol/L), *n* = 130	68 (62–74)	86 (70–101)	91 (60–122)	116 (80–151)^***^ vs. 1/^*^ vs. 2	0.009
Platelet at CT scan (10^9^/L)	342 (298–385)	339 (288–389)	362 (215–509)	318 (245–391)	0.91
P-procalcitonin at CT scan (μg/L), *n* = 128	0.21 (0.13–0.34)	0.48 (0.30–0.77)^*^ vs. 1	0.28 (0.07–1.11)	0.86 (0.40–1.88)^**^ vs. 1	0.010
D-dimer at CT scan (mg/L), *n* = 122	1.2 (0.9–1.6)	3.3 (2.4–4.6)^**^ vs. 1	6.1 (2.8–13.3) ^***^ vs. 1	9.6 (6.5–14.4)^***^ vs. 1/^***^ vs. 2	<0.0001
Troponin T at CT scan (ng/L), *n* = 110	10 (8–12) (*n* = 10)	28 (20–39)^***^ vs. 1 (*n* = 8)	30 (14–66) ^**^ vs. 1 (*n* = 2)	48 (30–77) ^***^ vs. 1/^*^ vs. 2	<0.0001
NT-proBNP at CT scan (ng/L), *n* = 66	750 (75–2,730) (*n* = 32)	1020 (531–1,977) (*n* = 13)	540 (94–3,087) (*n* = 7)	1820 (690–4,800) (*n* = 3)	0.096
Lowest PFI (PaO_2_/FiO_2_), *n* = 62	15.4 (10–21) (*n* = 31)	10.2 (8.5–12) (*n* = 26)	15.3 (8.9–21.6) (*n* = 4)	10.5 (8.4–12.5) (*n* = 7)	0.050^††^
PFI at CT scan (PaO_2_/FiO_2_), *n* = 56	22 (15–30) (*n* = 33)	13 (10–16) (*n* = 28)	23 (7–39) (*n* = 6)	20 (13–27) (*n* = 7)	0.054^††^
**Outcome data**					
Hospitalized at the end of study	7 (*n* = 3/42)	23 (*n* = 12/53)	8 (*n* = 1/12)	27 (*n* = 6/22)	0.09
ICU at CT scan	0	43 (*n* = 23/53)^*^ vs. 1	25 (*n* = 3/12)	73 (*n* = 16/22)^*^ vs. 1	<0.0001^†^
Intubated at CT scan	2 (*n* = 1/43)	43 (*n* = 23/53)^*^ vs. 1	25 (*n* = 3/12)	73 (*n* = 16/22)^*^ vs. 1	<0.0001^†^
Vasopressor treatment at CT scan	12 (*n* = 5/43)	49 (*n* = 24/49)^*^ vs. 1	55 (*n* = 6/11)	68 (*n* = 15/22)^*^ vs. 1	<0.0001^†^
Renal replacement therapy at CT scan	2 (*n* = 1/43)	21 (*n* = 11/53)^*^ vs. 1	8 (*n* = 1/12)	55 (*n* = 12/22)^*^ vs. 1	<0.0001^†^
ICU during stay	16 (*n* = 7/ 43)	68 (*n* = 36/53)^*^ vs. 1	67 (*n* = 8/12)	95 (*n* = 21/22)^*^ vs. 1	<0.0001^†^
60-day mortality	0% (*n* = 0/43)	26% (*n* = 14/53)^*^ vs. 1	17% (*n* = 2/12)	36% (*n* = 8/22)	<0.001^†^
Anticoagulant therapy at CT in deceased patients	–	86 (*n* = 12/14)	50 (*n* = 1/2)	75 (*n* = 6/8)	0.48^†^

*Demographics are expressed as median (interquartile range). Radiological and laboratory data are expressed as mean (± CI). Procalcitonin, D-dimer, troponin T, and NT-ProBNP were not normally distributed and log-transformed and are expressed as geometric mean (± CI). Comorbidities, radiology, and outcome data are expressed as %, n, affected subjects/total subjects in each group. For troponin T, NT proBNP, lowest PFI, and PFI at CT, the missing numbers (n) are shown*.

*Statistical analyses are based on analysis of variance (t-test) between groups. p <0.05 was considered significant*.

† *Pearson chi-squared*.

†† *Non-parametric Kruskal–Wallis test. Post-hoc with Fisher's test for non-parametric comparisons between mean rank. AMI, acute myocardial infarction; BMI, body mass index; CI, confidence interval; COPD, chronic obstructive pulmonary disease; CRP, C-reactive protein; CT, computed tomography; ICU, intensive care unit; N.A., not applicable; PE, pulmonary embolism; OSAS, obstructive sleep apnea syndrome; PFI, quotient between arterial partial oxygen pressure and fraction of inspired oxygen; NT-proBNP, N-terminal pro brain natriuretic peptide; RV, right ventricular*.

* *p < 0.05*,

** *p < 0.01, and*

*** *p < 0.001*.

### Laboratory Measurements

CRP was determined by immuno-turbidimetric analysis using Cobas (Diagnostics, Roche, GmbH, Mannheim, Germany; normal value <5.0 mg/L). White blood cell (WBC) count was analyzed by fluorescence using Sysmex XN (Sysmex Corporation, Hyogo 651-0073, Japan; reference value 3.8–8.8 × 10^9^/L). Hemoglobin was analyzed by photometry using Sysmex XN (Sysmex Corporation, Hyogo 651-0073, Japan; normal range 134–170 g/L for men and 117–153 g/L for women). Platelet was analyzed using impedance and flow cytometry (Sysmex Corporation, Hyogo 651-0073, Japan; reference value 165–387 × 10^9^/L for women and 145–348 × 10^9^/L for men). Plasma creatinine (anticoagulated with Li-heparinate) was analyzed using an enzymatic photometric method (Cobas, Diagnostics, Roche, GmbH, Mannheim, Germany; reference value <100 μmol/L for men and <90 μmol/L for women). Procalcitonin was analyzed using the Cobas 8000/e602 system (Diagnostics, Roche, GmbH, Mannheim, Germany; reference value < 0.05 μg/L for women and 0.08 μg/L for men). D-dimer was analyzed using the Sysmex CS-5100 and CS-2500 (Siemens Healthcare Diagnostics, Erlangen, Germany) with monoclonal antibodies in diluted samples (analyzing interval 0.19–35.20 mg/L, inter-assay coefficient of variation <11.25%, reference value <0.5 mg/L below 50 years and <0.7 mg/L above 70 years). Troponin T and NT-proBNP were analyzed in plasma samples using the Cobas e601, e602 system (Diagnostics, Roche, GmbH, Mannheim, Germany; reference value <15 ng/L for troponin T, and <300 ng/L below 50 years and <400 ng/L for 50–75 years for NT-pro BNP).

The fraction of inspired oxygen was measured directly on ventilators or calculated from the oxygen flow and administration method. The PaO_2_ was obtained from the blood gas analysis (ABL800 FLEX, Triolab AB, Mölndahl, Sweden; PFI reference >50 kPa).

### Statistical Analysis

Anthropometric data are presented as median (interquartile range). Radiological and laboratory parameters are presented as mean ± 95% confidence interval (CI) in Tables 1, 2. Normal distribution was achieved by log-transformation of procalcitonin, D-dimer, troponin T, and NT-proBNP values and are presented as geometric mean ± CI. Student's *t*-test was used for comparison of means, when the distribution was normal. In non-normal distribution, the Mann–Whitney *U*-test was used for comparisons (lowest PFI during hospitalization). For multiple variables, the analysis of variance (ANOVA), analysis of co-variance (ANCOVA), and *post-hoc* Fisher's test were used, when the distribution was normal, while Kruskal–Wallis test and *post-hoc* multiple comparisons of mean ranks test were applied when the distribution was non-normal (Table 2). Pearson's *x*^2^ test was used for the comparison of categorical variables. Single odds ratios (ORs) for the PE event and 60-day mortality were calculated for certain variables of interest (Table 3), and a multiple OR calculation was performed. The upper and lower (for hemoglobin and platelet count) interquartile values in all 130 patients were labeled as cutoff values for the OR calculation (Table 3). A *p* < 0.05 was considered significant. Statistical analyses were performed using Statistical Stat Soft, version 10 (Tulsa, Ok, USA).

**Table 3:** Odds ratios for certain variables to PE event and 60-day mortality.

**Variable**	***N***	**Above or below median laboratory value**	**Odds ratio PE (± 95% confidence interval)**	***p*-value**	**Odds ratio 60-day mortality (± 95% confidence interval)**	***p*-value**
Kidney disease	130		0.69 (0.07–6.6)	0.74	7.4 (1.1–48.1)	0.035
CRP max (mg/L)	130	≥343	2.7 (1.1–6.2)	0.024	3.1 (1.2–7.9)	0.020
White blood cell count (10^9^/L)	130	≥12.4	2.7 (1.1–6.2)	0.024	3.8 (1.5–9.7)	0.005
Hemoglobin (g/L)	130	≤ 96	2.8 (1.2–6.7)	0.017	2.6 (1.0–6.6)	0.051
P-creatinine (μmol/L)	130	≥102	2.8 (1.2–6.7)	0.017	8.1 (3.1–21.7)	<0.0001
Procalcitonin (μg/L)	127	≥0.92	2.6 (1.1–6.1)	0.034	3.3 (1.3–8.5)	0.014
P-thrombocytes	130	≤ 227	1.3 (0.5–3.2)	0.53	2.1 (0.8–5.3)	0.14
D-dimer (mg/L)	122	≥7.2	5.0 (2.0–12.0)	0.0003	4.0 (1.5–10.6)	0.005
Troponin-T (10^9^/L)	110	≥45	2.7 (1.1–6.6)	0.034	13.0 (4.4–38)	<0.0001
PFI lowest (PaO_2_/FiO_2_)	62		0.47 (0.13–1.7)	0.24	6.0 (1.7–21)	0.003
ICU stay	130		7.1 (2.5–20)	<0.0001	7.5 (2.1–27)	<0.001
Vasopressor treatment at CT	125		3.8 (1.6–8.8)	0.001	6.5 (2.3–18)	0.0001
Renal replacement therapy at CT	130		4.3 (1.7– 11.0)	0.002	5.6 (2.1–15.0)	0.001
Widespread parenchymal abnormalities	130		1.4 (0.6–3.2)	0.39	10.6 (2.3–48)	<0.0001

*CRP, C-reactive protein; CT, computed tomography; ICU, intensive care unit; PE, pulmonary embolism; PFI is the quotient between arterial partial oxygen pressure and the fraction of inspired oxygen*.

## Results

### General Patient Data

This study included 130 patients with a positive COVID-19 RT-PCR and clinically suspected to have PE [35 women and 95 men, median age 57 years (interquartile range 51–64)]. The patients were investigated with CTPA on average 21 days after symptom onset (range 1–55 days) and comprised 20% of all 640 hospitalized patients. PE was confirmed in 34 patients, which represents 26% of patients who underwent CTPA and 5.3% of all hospitalized COVID-19 patients. Anthropometric data for all individuals are presented in Table 1. All but 8 of the 34 (24%) patients were on anticoagulant therapy prior to the diagnosis of PE. Patients with PE had a longer time delay from first symptoms to CT scan (*p* < 0.05, Table 1), but no differences in days of hospitalization prior to CTPA (Table 1). Of all patients, 15 out of 130 (12%) were examined with CT scan at the day of admission, with confirmed PE in 13% of cases.

**Figure 6:**
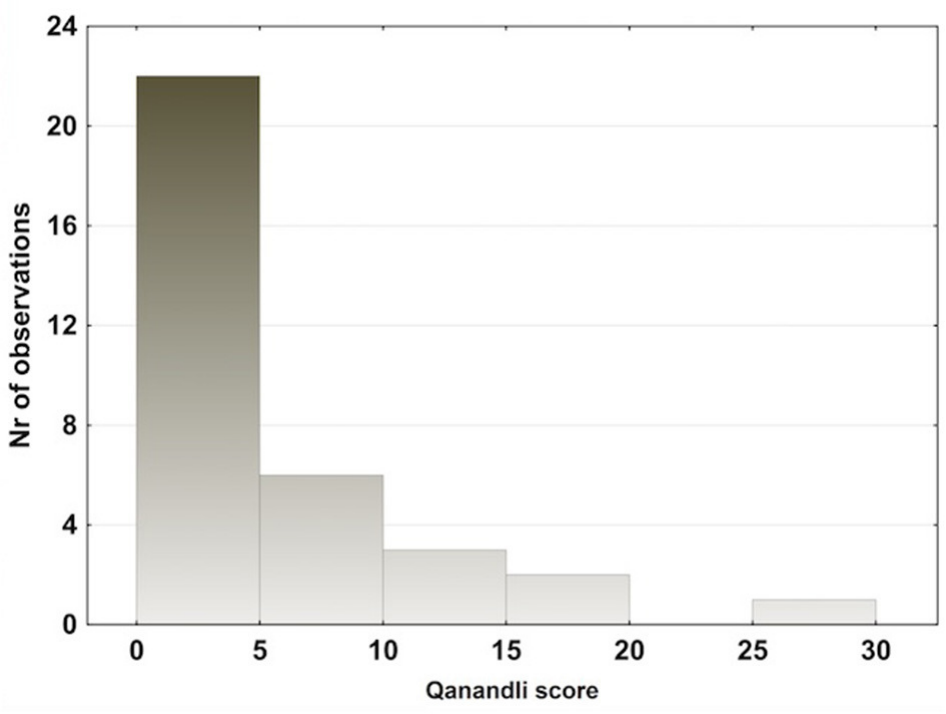
Qanadli score in the pulmonary embolism (PE) group. Sixty-five percent of the PE patients had a Qanadli score between 0 and 5, indicating small peripheral emboli in a large part of the PE cohort.

**Figure 7:**
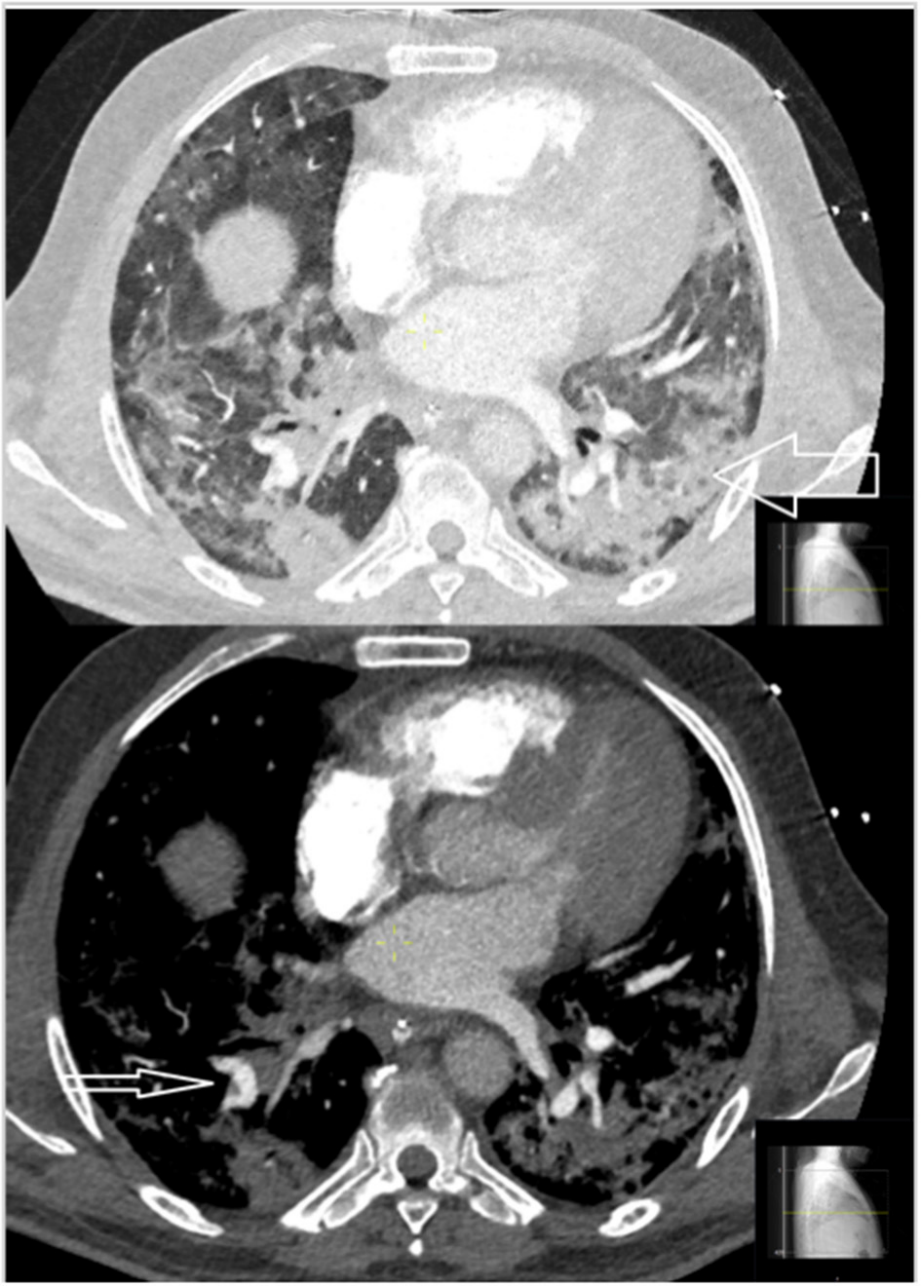
CT images of two different patients with COVID-19. A 58-year-old male with widespread parenchymal abnormalities and consolidations (large arrow) and small peripheral emboli (small arrow). Two-thirds (22/34) of the PE patient group were classified as having widespread parenchymal abnormalities.

**Figure 8:**
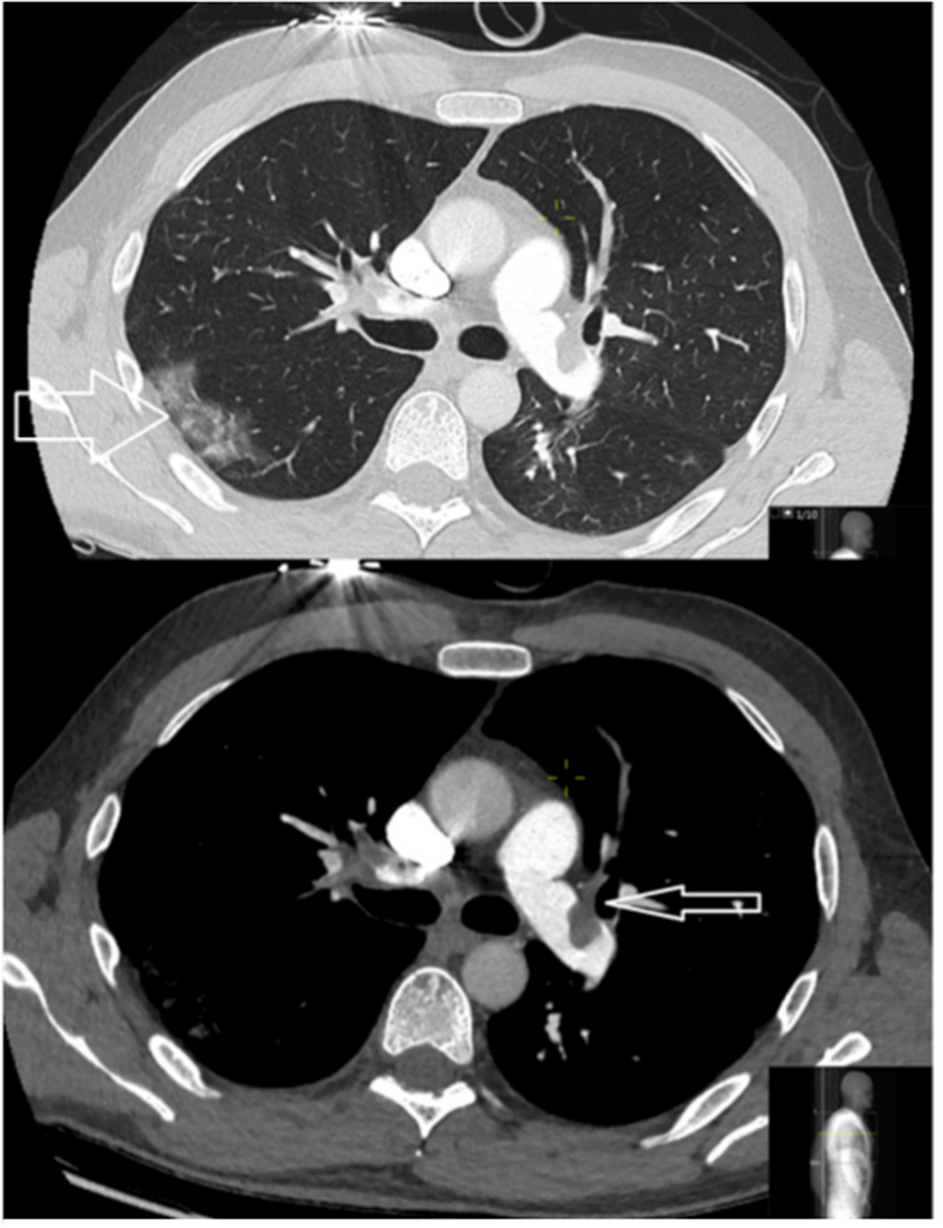
CT images of two different patients with COVID-19. A middle-aged male with limited peripheral ground glass opacities (large arrow) and large central emboli (small arrow).

**Figure 9:**
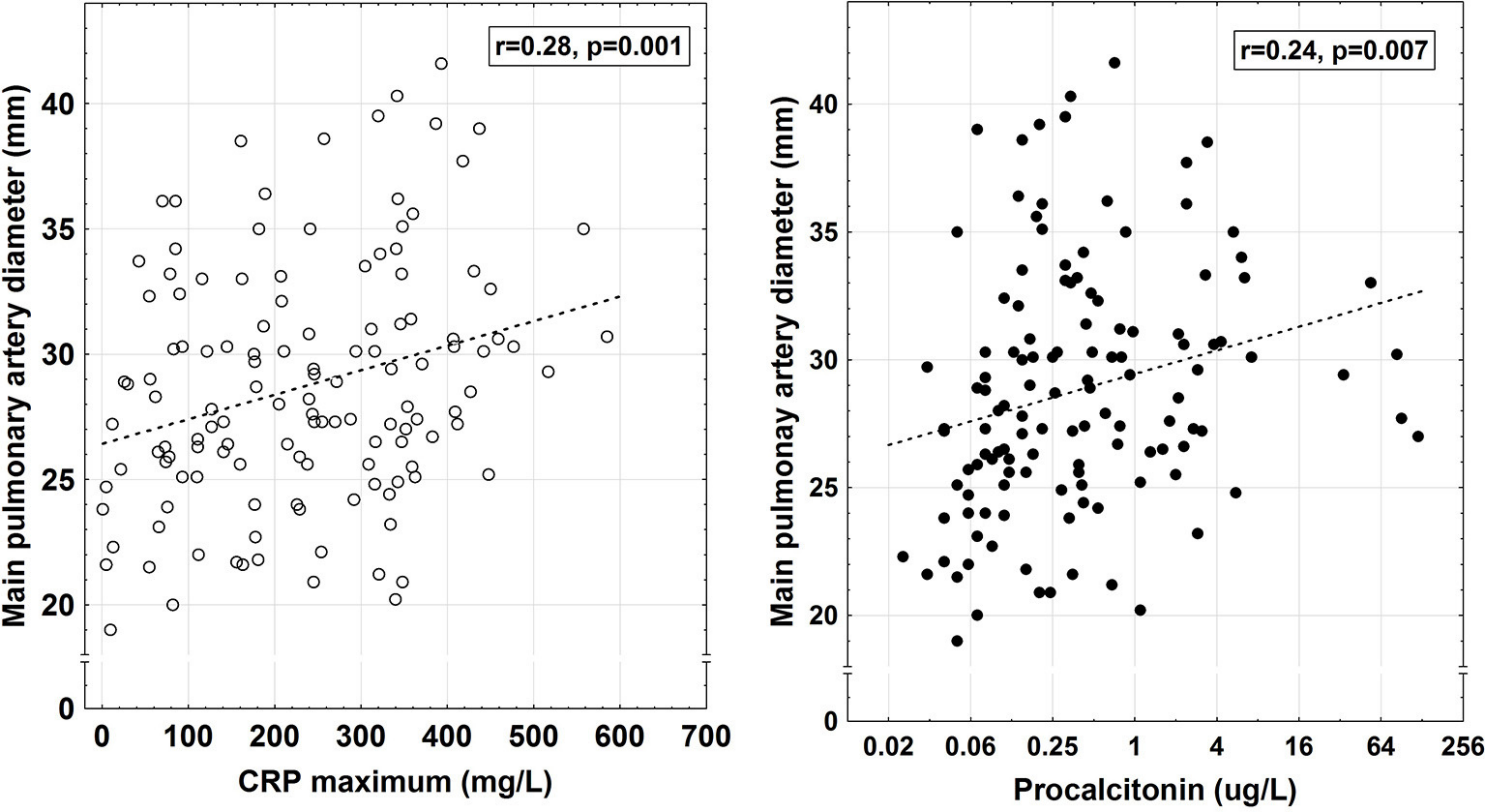
Correlation between the main pulmonary diameter on CT and maximum C-reactive protein [CRP; unfilled circle; *y* = 26.4208 + 0.098*x, r* = 0.28, *p* = 0.001, dashed line (…), *n* = 130] or procalcitonin levels [filled circle; *y* = 29.444 + 0.463*x, r* = 0.24, *p* = 0.007, dashed line (…), *n* = 127] in hospitalized COVID-19 patients.

Segmental/sub segmental, rather than central/lobular, emboli were found in 22 of the 34 patients (65%; Qanadli score ≤ 5; Figures 6–8). Increased main pulmonary artery diameter correlated positively with max CRP (*r* = 0.28, *p* = 0.001, and *n* = 130) and procalcitonin (*r* = 0.24, *p* = 0.006, and *n* = 127) in the entire cohort (Figure 9).

Pulmonary artery diameter at CT did not differ between patients with or without PE (Table 1). A tendency for increased right ventricular diameter was present in the PE group (*p* = 0.06, Table 1). Max CRP together with CRP, WBC counts, P-creatinine, procalcitonin, D-dimer, and troponin T all obtained at CT timepoint were significantly higher while hemoglobin was lower in patients positive CT for PE, compared to those without PE (Table 1). The prevalence of intubation at CT, ICU stay, vasopressor treatment, and renal replacement therapy were higher in the PE group (Table 1). D-dimer correlated positively with length of hospitalization in the group without PE (*r* = 0.397, *p* < 0.0001, and *n* = 90), but not in the PE group (*r* = −0.09).

### Patients With or Without Widespread Parenchymal Abnormalities

**Figure 10:**
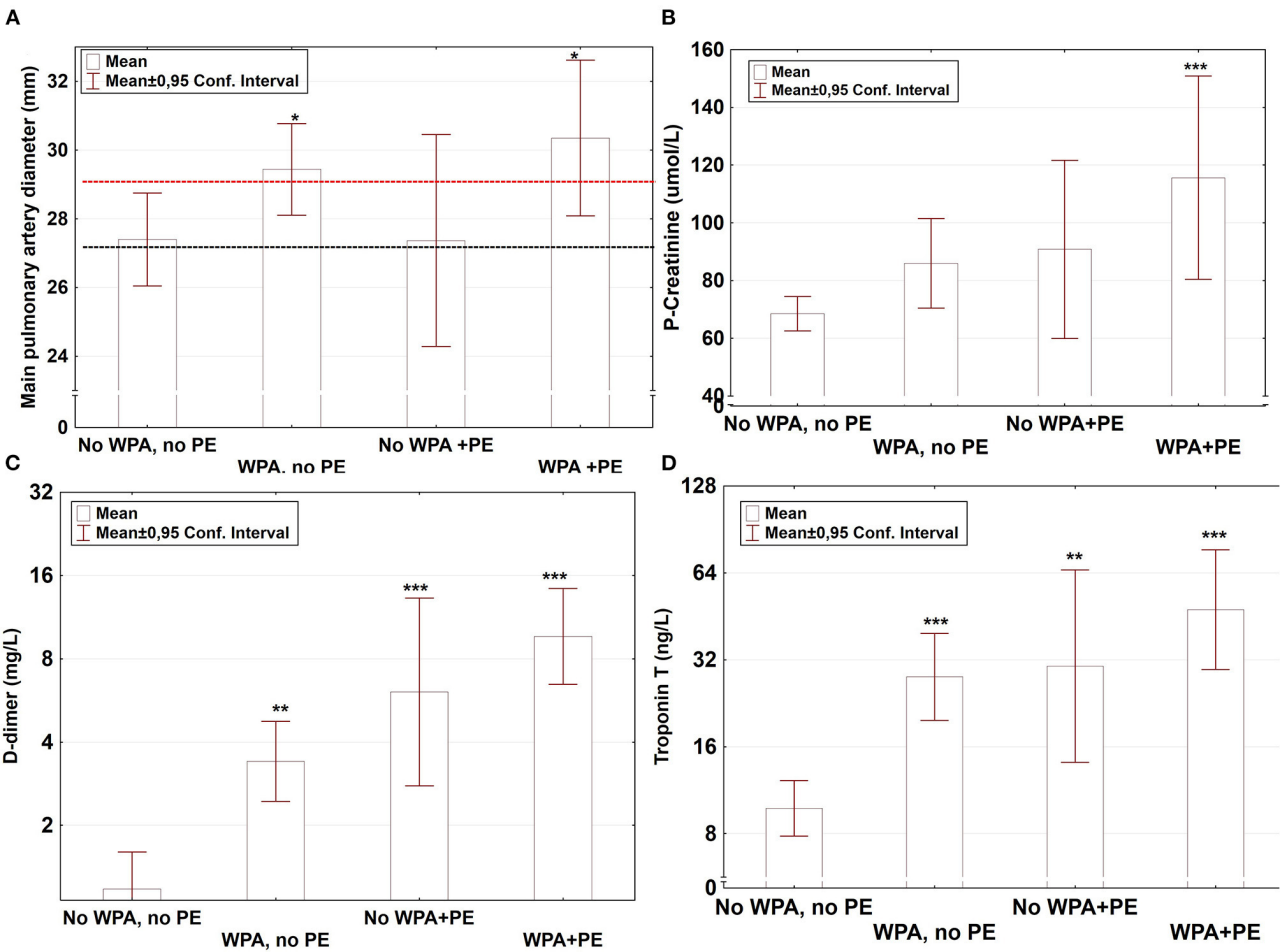
Main pulmonary artery diameter (mm), P-creatinine (μmol/L), D-dimer (mg/L), and troponin T (10^9^/L) in the four different groups. **(A)** Main pulmonary artery diameter (mm), **(B)** P-creatinine (μmol/L), **(C)** D-dimer (mg/L), and **(D)** troponin T (10^9^/L). Bars represent mean values ± 95% confidence intervals in the different groups. (1) No widespread parenchymal abnormalities (WPA), no pulmonary embolism (PE); (2) WPA, no PE; (3) No WPA+PE; and (4) WPA+PE. For main pulmonary artery diameter, the red and black dashed lines represent 90% cutoff values in men and women, respectively, from a large reference material of 3,171 men and women (12).

Of the 130 patients, 76 (58%) were radiologically classified as having WPA (Table 2). No major differences in comorbidities were found between the groups with or without WPA. However, patients with WPA had significantly higher max CRP, WBC count, P-creatinine, procalcitonin, D-dimer, and troponin T levels (Figure 10) and lower hemoglobin levels than the group without PE and without WPA (Table 2). Dividing the material into two groups with or without WPA, the WPA group had higher D-dimer levels [mean ± CI: 4.6 (3.1–6.1) (*n* = 70) vs. 1.7 (1.2–2.3) (*n* = 52) mg/L; *p* < 0.0001, *n* = 122, ANOVA *t*-test]. In *post-hoc* analysis, higher D-dimer was present in the WPA group with PE compared with WPA without PE (*p* < 0.001, Table 2). Pulmonary artery diameter was greater in patients with WPA, compared to those without WPA (*p* < 0.05, *post-hoc* Fisher's test, Table 2, Figure 10).

A higher proportion of patients had standard/prophylactic anticoagulant therapy in the WPA without PE compared to the WPA with PE group (30 vs. 5%) (Table 2). Patients with WPA with or without PE had a tendency to a lower oxygenation index during hospitalization (*p* = 0.05, Kruskal–Wallis test, Table 2).

### Sixty-Day Mortality

No deaths occurred in the group without both PE and WPA. In the WPA groups, with and without PE, the mortality rate was 36 and 26%, respectively (*p* < 0.001, Table 2). The two patients without WPA + PE who died had Qanadli scores of 7 and 27 (highest in the entire group). A wider main pulmonary artery diameter, adjusted for weight, was found in non-surviving patients compared to surviving patients [width 30.6 mm (28.7–32.3) vs. 28.3 mm (27.4–29.2); *p* = 0.037, *n* = 125, ANCOVA, test]. Right ventricular diameter was not related to mortality.

### Odds Ratio Analyses

Univariate OR analysis of certain variables for PE and 60-day mortality are shown in Table 3.

In multiple OR testing (with the variables CRP maximum, WBC count, hemoglobin P-creatinine, D-dimer, procalcitonin, and ICU during stay), PE was related to D-dimer >7.2 mg/L, [OR = 4.1 (CI = 1.4–12.0)] and ICU stay [OR = 5.6 (CI = 1.5–21)] (*p* = 0.0004, *n* = 120). In another multiple OR testing model (with the laboratory variables CRP maximum, WBC count, hemoglobin, P-creatinine, platelet, D-dimer, and troponin T), troponin T > 40 × 10^9^/L [OR = 8.1 (CI = 1.9–35)], D-dimer >7.2 mg/L [OR = 5.2 (CI = 1.3–20.1)], and P-creatinine >102 μmol/L [OR = 4.9 (CI = 1.3–20)] (*p* = 0.0001, *n* = 106) were associated with 60-day mortality. In addition, in the entire cohort, WPA [OR = 8.5 (Cl = 1.8–41)] and P-creatinine >102 μmol/L [OR = 5.7 (CI = 2.0–16.1)] were significantly associated with 60-day mortality (*p* < 0.0001, *n* = 130), while the presence of PE was not.

## Discussion

The present study describes radiological findings in hospitalized COVID-19 patients who underwent CTPA for suspected PE and assesses possible associations with disease severity and mortality. PE was confirmed in 26% of cases. Patients with PE were more often treated in the ICU and required mechanical ventilation. The majority of the pulmonary emboli were small, with a sub-segmental distribution. Patients with PE had higher D-dimer, troponin T, WBC count, CRP, and P-creatinine levels and lower hemoglobin levels compared to patients without PE.

The highest mortality in our study was observed in the patient phenotype with WPA combined with PE and higher CRP levels. Furthermore, the mortality was higher in patients with WPA and lower PFI. These findings reflect a more severe COVID-19 disease and are in agreement with the criteria defining severity in ARDS (13).

The frequency of PE in the present study is in line with data reported from other European countries (4, 5). In the Netherlands, thromboembolic complications were reported in 57 of 184 (31%) patients with COVID-19 ARDS and treated in ICU, and the lung was the most common localization of thromboembolism (25%) (5). In a French study, in which all consecutive patients with COVID-19 performed CTPA at admission, PE was diagnosed in 14.2% of the patients (14). Our cohort had a higher frequency of PE than another study that also reported a lower incidence of ICU admissions, suggesting that fewer patients were critically ill (15). In a recent review, the prevalence of PE in hospitalized COVID-19 patients was reported to be 3.5% in non-ICU and 13.7% in ICU patients, with an overall incidence of venous thromboembolism of 14% across the studies included in their analysis (6).

Prolonged immobilization in the ICU increases the risk of thromboembolic disease. However, the occurrence of PE observed in our study is higher than previous reports on non-COVID ICU patients (16) or on critically ill patients (17). Accordingly, within an ICU setting, Helms et al. (16) also found significantly more thrombotic events in COVID-19 ARDS, compared to non-COVID-19 ARDS, mainly represented by PE (14 vs. 2%). The difference reported between studies on PE prevalence in COVID-19 also mirrors the difficulty during the COVID-19 pandemics to standardize PE screening (18).

Autopsy data on COVID-19 patients have clearly revealed distinct vascular findings in the lungs, with widespread thrombosis and microangiopathy nine times more frequent than in patients with ARDS secondary to influenza (19). In an interesting study by Diehl et al. (7), the authors suggest that endothelial damage may play an important role in the pathogenesis of COVID-19 respiratory failure, since the SARS-COV2 receptor (ACE2) is highly expressed on the surface of endothelial cells. The viral infection may damage the endothelial cells and may then trigger the activation of coagulation. The authors demonstrated the presence of endothelial lesions in COVID-19 ARDS, by measuring increased levels of circulating endothelial cells (marker of endothelial lesion) together with raised D-dimers (marker of thrombosis) and found an association with increased physiological dead space and ineffective ventilation. Furthermore, curative anticoagulation could prevent COVID-19 coagulopathy and endothelial lesion (20).

The study by Fauvel et al. (21) strengthens the strong interaction of inflammation and coagulopathy in COVID-19. In our study, WPA were also associated with a high inflammatory state, mirrored by high CRP, WBC count, procalcitonin, and D-dimer. The increase of D-dimers may reflect microthrombi formation in COVID-19, and indeed, reported histopathological observations revealed the presence of alveolar fibrin deposits in pulmonary samples (22).

The interpretation of increased D-dimer levels in COVID-19 is a complex issue and has been debated in many studies. There is a technical difficulty comparing results from different assays expressed in different units (23). In line with our findings, some studies have shown D-dimer to be a marker of COVID-19 disease severity and a predictor of mortality (24, 25). Other researchers showed that higher D-dimer levels were associated with a higher risk of PE (21, 26, 27). Furthermore, some authors suggest the use of a predefined D-dimer level as a tool in PE diagnosis, with the cutoff adjusted for COVID-19 (18). In addition, there have been attempts to identify a D-dimer threshold for risk stratification and support for enhanced anticoagulation (28).

The optimal strategy for prevention of thromboembolic events in critically ill COVID-19 patients is uncertain. Clinical guidelines suggest standard dose LMWH in hospitalized COVID-19 patients and intermediate doses to be considered in ICU patients with high risk (29). Recent studies have reported benefits of anticoagulation therapy before hospitalization (21, 30) and guidelines suggest the same (31). Some studies have shown lower mortality rates in severe COVID-19 treated with therapeutic doses of LMWH compared with prophylactic/intermediate doses (32, 33). According to Tang et al., neither prophylactic nor intermediate doses of LMWH in patients with very severe COVID-19 are associated with a better outcome (34). According to Sadeghipur, the use of intermediate doses, compared with standard doses, did not result in lower incidence of embolism or mortality in ICU patients (35).

From a morphological point of view, the analysis of the PE distribution in our cohort revealed the presence of both central/lobular and segmental/sub-segmental emboli, with a predominance of the latter. Increased main pulmonary artery diameter was associated with WPA and 60-day mortality. The main pulmonary diameter has been regarded as a sign of pulmonary hypertension, and a diameter >29 mm is often used as a cutoff in men (12, 36). However pulmonary hypertension is also seen in non-COVID-19-associated ARDS and is multifactorial, with factors including hypoxia and hypercapnia, severity of the parenchymal disease, and possibly microembolism.

Higher troponin T levels were associated with an increased 60-day mortality rate. Other studies have reported the same finding (37). This reflects myocardial damage that may be caused by severe infection, hypoxia, cytokine storm, and cardiovascular microthrombosis or embolism (38, 39). In the study by Goudot et al., the high-sensitivity cardiac troponin was shown to be the best predictor of ICU referral for COVID-19 patients (40). Furthermore, the association of high levels of high-sensitivity cardiac troponin, D-dimer, and right ventricle dilatation emphazises the hypothesis of underlying microthrombosis in COVID-19, causing increased right ventricle afterload, dilatation, and some degree of myocardial injury (40).

The lowest hemoglobin levels were found in patients with WPA and PE. Low hemoglobin has also been reported frequently in the ICU and in critically ill COVID-19 patients (41). We believe that lower hemoglobin levels in COVID-19 patients could be explained by longer hospitalization and by red blood cell destruction in presence of microthrombosis.

In the present study, P-creatinine was also associated with 60-day mortality in the multiple OR analysis. This finding may reflect kidney injury of thromboembolic origin in COVID-19 disease (42) and has previously been correlated with increased D-dimers levels (43). Whether iodinated contrast is more toxic in COVID-19 is not known.

One strength of the present study is the prospective collection of patients with a positive virus test from regular wards, high-dependency units, and ICUs. Two experienced radiologists validated the CT findings independently, and thin slices allowed us to detect both small and large emboli and lung parenchymal disease. The present study comprises a relatively small sample of patients, but our data highlight possible morphological and laboratory markers of COVID-19 disease severity. Of the variables considered in our study, D-dimer and troponin T best predicted the disease severity.

One major limitation of this study is that patients were chosen by convenience sampling. Only patients with a clinical suspicion of PE and stable enough to be transported to the radiology department were investigated by CTPA and included in the study. Thus, some COVID-19 patients were missed and even the occurrence of PE might have been underestimated. Therefore, our findings may not be generalized to the entire population of COVID-19 patients. Radiological limitations include respiration artifacts, which, in combination with widespread consolidations, could make the detection of small peripheral PE more difficult. These technical difficulties may lead to underestimation of the real incidence of PE in patients with WPA. However, we do not believe that this altered the major findings in the study.

In conclusion, in our population, clinically suspected pulmonary embolism was confirmed by radiology in 26% of cases. However, widespread pulmonary infiltrates (WPA) were even more common (66%). D-dimer was confirmed as the superior laboratory parameter for prediction of risk for pulmonary embolism. Pulmonary embolism contributed to mortality risk in COVID-19 patients with widespread parenchymal abnormalities. Most thrombi were small and could be easily missed. Elevated CRP, D-dimer, troponin-T, P-creatinine, and enlarged pulmonary artery were associated with a worse outcome and may mirror a more severe systemic disease. A liberal approach to radiological investigation should be recommended at clinical deterioration, when the situation allows it. Although CTPA added to risk assessment, CT imaging even without intravenous contrast to assess the severity of pulmonary infiltrates in combination with clinical laboratory parameters might be of value to predict outcome. Better radiological techniques with higher resolution could improve the detection of microthromboses. This could influence anticoagulant treatment, preventing clinical deterioration.

## Data Availability Statement

The datasets presented in this study can be found in online repositories. The names of the repository/repositories and accession number(s) can be found at: 10.6084/m9.figshare.13299164.

## Ethics Statement

The studies involving human participants were reviewed and approved by Karolinska University Hospital, Sweden. Written informed consent for participation was not required for this study in accordance with the national legislation and the institutional requirements.

## Author Contributions

FJ, MOB, MB, SN, and AK were involved in the concept and design of the study. FJ, AK, MOB, SN, and AS performed the data analysis. FJ, MOB, MS, AS, MB, FS, SN, and AK contributed to interpreting the data. The first draft was written by FJ and AK. All co-authors were engaged in revising the final version of the manuscript and provided final approval of the version to be published.

## Conflict of Interest

The authors declare that the research was conducted in the absence of any commercial or financial relationships that could be construed as a potential conflict of interest.

## Abbreviations

ARDS: Acute respiratory distress syndrome
ACE2: Angiotensin-converting enzyme
BMI: Body mass index
CT: Computed tomography
CI: Confidence interval
CRP: C-reactive protein
CTPA: Computed tomography pulmonary angiogram
ICU: Intensive care unit
LMWH: Low-molecular-weight heparin
OR: Odds ratio
PACS: Picture archiving and communication system
PaO_2_/FiO_2_: Quotient between arterial partial oxygen pressure/oxygen inspired fraction
PE: Pulmonary embolism
NT-proBNP: N-terminal pro brain natriuretic peptide
RIS: Radiological information system
RT-PCR: Real-time polymerase chain reaction
SARS-CoV-2: Severe acute respiratory syndrome coronavirus-2
WPA: Widespread parenchymal abnormalities
WBC: White blood cell.
